# LINE-1 and SINE-B1 mapping and genome diversification in *Proechimys* species (Rodentia: Echimyidae)

**DOI:** 10.26508/lsa.202101104

**Published:** 2022-03-18

**Authors:** Simone Cardoso Soares, Eduardo Schmidt Eler, Carlos Eduardo Faresin e Silva, Maria Nazareth Ferreira da Silva, Naiara Pereira Araújo, Marta Svartman, Eliana Feldberg

**Affiliations:** 1 Pós-Graduação em Genética, Conservação e Biologia Evolutiva, Instituto Nacional de Pesquisas da Amazônia, Manaus, Brazil; 2 Laboratório de Genética Animal (LGA), Instituto Nacional de Pesquisas da Amazônia, Manaus, Brazil; 3 Universidade do Estado do Amazonas, Manaus, Brazil; 4 Coleção de Mamíferos, Instituto Nacional de Pesquisas da Amazônia, Manaus, Brazil; 5 Departamento de Genética, Ecologia e Evolução, Universidade Federal de Minas Gerais, Belo Horizonte, Brazil; 6 Instituto Federal de Educação, Ciência e Tecnologia de Rondônia campus Jaru, Jaru, Brazil

## Abstract

This study aimed to understand the impact of LINE-1 and SINE-B1 retroelements on the architecture and karyotypic diversification of five rodent species of the genus *Proechimys* from different regions of the Amazon. Karyotype comparisons were performed using fluorescent interspecific in situ hybridization. The L1 and B1 retroelements showed a non-random arrangement and a conserved pattern when the genomes of the five species of *Proechimys* were compared, including the two cytotypes of *Proechimys guyannensis*. The signal homeology among the chromosomes and the degree of similarity among the formed clusters indicate rearrangements such as fusion/fission, and demonstrates that these retroelements can behave as derived characters shared in *Proechimys*. The differentiated distribution and organization of these retroelements in the karyotypes and in the chromosomal fiber, respectively, may represent a strong indication of their role as generating sources of karyotypic diversity in the genus *Proechimys* and provide insights into the evolutionary relationships between taxa.

## Abstract (precis)

Signal distribution of L1 and B1 among chromosomes in five species of *Proechimys* may represent a strong indication of their role in karyotype diversity of this speciose Neotropical rodent.

## Introduction

Transposable elements (TEs) are repetitive nucleotide sequences that are dispersed throughout the genome in euchromatin and heterochromatin regions. This distribution varies according to the taxon and can represent 3–50% of the genome depending on the species (Capy et al, 2000; Levin & Moran, 2011; Vandewege et al, 2016).

During transposition, TEs can be inserted into genes or regulatory elements of genes, triggering chromosomal rearrangements, as suggested by Araújo et al (2017) for Akodontini rodents, contributing to genetic diversity and even impairing gene function (Paço et al, 2014). Some studies have shown the influence of TEs in the genome size of species, such as the difference in the number of copies of TEs in the genomes of *Drosophila melanogaster* and *Aedes aegypti* (Kidwell & Lisch, 2001), and alterations in gene expression and/or accentuated activity in the development of cancer in mice and humans (Faulkner et al, 2009; Tubio et al, 2014). Other evidence suggests that these elements are active in cultured mouse neuronal precursor cells and in the brains of mice (Muotri et al, 2005; Coufal et al, 2009).

The retroelements LINE-1 (L1) and SINE-B1 (B1) are repetitive, interspersed gene sequences that represent a predominant part of the genome of humans and mice (Boyle et al, 1990; Richardson et al, 2015). L1 comprises long and dispersed elements, with sizes varying between 6 and 7 kb, and are considered autonomous because they encode proteins necessary for their own mobilization (Kajikawa & Okada, 2002; Denli et al, 2015). B1 are non-autonomous short dispersed elements, with a length of ∼150 bp, that use the LINEs mechanism for their mobilization (Kajikawa & Okada, 2002; Veniaminova et al, 2007).

L1 and B1 comprise about 18% and 2.7% of the mouse genome, respectively. Functional studies in cultured cells of mice revealed 3,000 L1 sequences that are compatible with retrotransposition. However, the number of active B1 sequences remains unknown because of the difficulty in identifying subfamilies given the short length of the sequences (Dewannieux & Heidmann, 2005; Richardson et al, 2015; Yang et al, 2019).

In addition to activity in dynamic, sometimes deleterious processes, such as transcriptional regulation, epigenetic control, cell differentiation, and reprogramming, retrotransposons have also been associated with the structuring and organization of chromosomes (Töhönen et al, 2015; Razali et al, 2019). LINEs, for example, appear to be interconnected with chromatin remodeling, and accumulate in heterochromatin rich regions, such as the centromere and sex chromosomes, contributing to karyotype diversity (Kuznetsova et al, 2006; Meyer et al, 2016; Sotero-Caio et al, 2017). Furthermore, because of their significant contribution to mutations, LINEs play an expressive role in the adaptation and evolution of populations and species (Kazazian, 2004; Jurka et al, 2011).

*Proechimys* is a speciose and chromosomally variable Neotropical rodent. The taxonomic identification of *Proechimys* species is problematic due in part to inter- and intraspecific morphological overlaps, with many species presenting subtle variations in their characteristic morphological traits (Patton et al, 2000; Eler et al, 2020). Cytogenetic studies have shown great chromosomal variability in this group of rodents, with diploid numbers ranging from 14 to 62, and FN (number of autosome arms) ranging from 18 to 80. Intraspecific karyotype diversity in sympatry has also been reported, making it difficult to understand the relationships among species (Weksler et al, 2001; Eler et al, 2012; Amaral et al, 2013). Eler et al (2020) presented new karyotypes for *Proechimys* and expanded the geographical distribution of some cytotypes, attesting to the great karyological diversity of this genus. Given this scenario, the current study seeks to investigate the role of TEs in the karyotypic evolution of *Proechimys*.

Repetitive sequences such as TEs can trigger molecular variations in populations causing phenotypic consequences for individuals, and may be involved in the speciation process (Razali et al, 2019; Schrader & Schmitz, 2019). According to Ricci et al (2018), TEs significantly influence the evolution of genomes, particularly because of their involvement in chromosomal rearrangements and the correlation of their activity in mammal speciation.

In this study, we mapped the transposable retroelements L1 and B1 in the chromosomes of five species of the spiny rat *Proechimys* for the first time. By comparing results from different regions of the Amazon, we assess the impact of these retroelements on genome architecture and karyotype diversification of this taxon.

## Results

**Figure 1.**
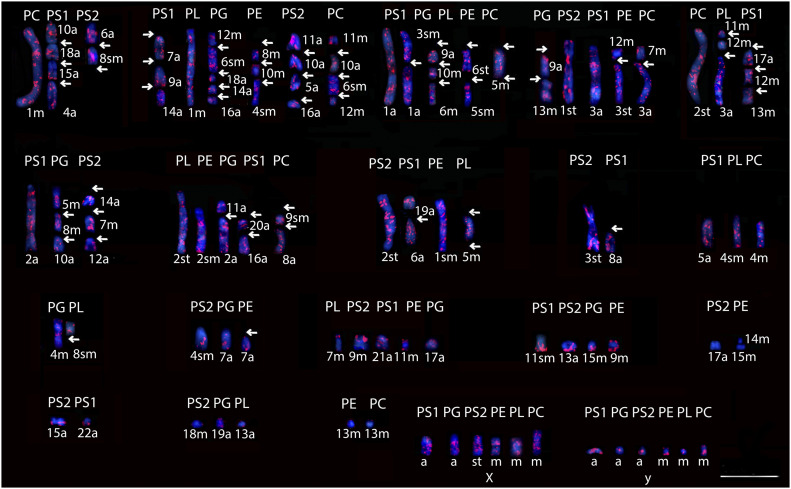
Distribution of the retroelement LINE-1 in species of *Proechimys *spp. PS1, *P. guyannensis* (2n = 46); PG, *P. gardneri* (2n = 40); PS2, *P. guyannensis* (2n = 38); PE, *P. echinothrix* (2n = 32); PL, *P. longicaudatus* (2n = 28); PC, *P. cuvieri* (2n = 28); m, metacentric; sm, submetacentric; st, subtelocentric; a, acrocentric. The arrows indicate the regions where possible rearrangements in the chromosomes of the different species occurred (Fig S1). 10.0 µm. Source data are available online for this figure.

**Figure 2.**
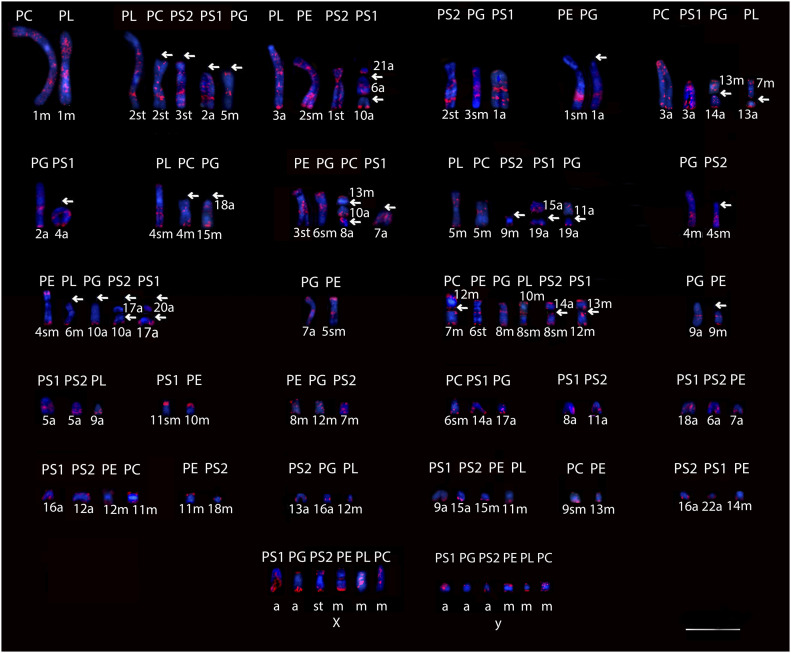
Distribution of the retroelement SINE B1 in *Proechimys* spp. PS1, *P. guyannensis* (2n = 46); PG, *P. gardneri* (2n = 40); PS2, *P. guyannensis* (2n = 38); PE, *P. echinothrix* (2n = 32); PL, *P. longicaudatus* (2n = 28); PC, *P. cuvieri* (2n = 28); m, metacentric; sm, submetacentric; st, subtelocentric; a, acrocentric. The arrows indicate the regions where possible rearrangements in the chromosomes of the different species occurred (Fig S2). 10.0 µm. Source data are available online for this figure.

The analysis of the karyotypes of the five species of *Proechimys* (*Proechimys guyannensis* [PS1, 2n = 46], *Proechimys guyannensis* [PS2, 2n = 38], *Proechimys gardneri* [PG, 2n = 40], *Proechimys echinothrix* [PE, 2n = 32], and *Proechimys longicaudatus* [PL, 2n = 28] and *Proechimys cuvieri* [PC, 2n = 28]) allowed us to visualize the distribution of the retrotransposable elements LINE-1 and SINE-B1 in each chromosomal pair and comparison of the pattern of these retroelements between species (Figs 1 and 2).

**Figure S1.**
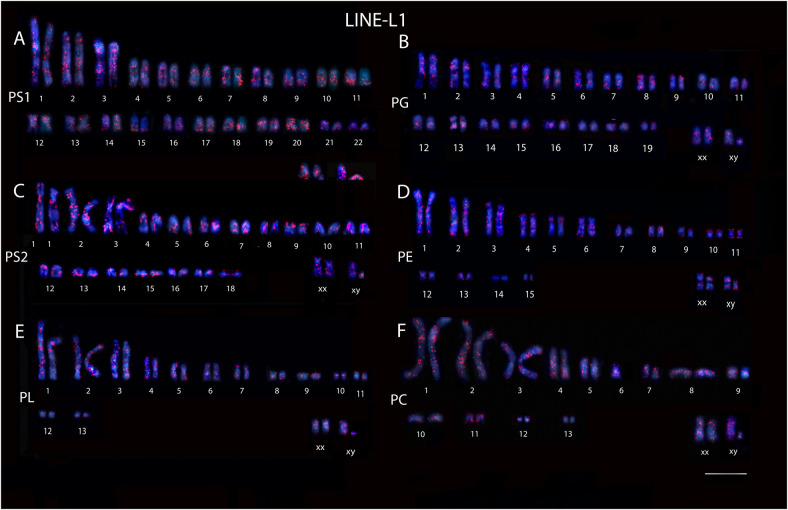
Karyotype of the five species of *Proechimys*, male and female, including two cytotypes of *P. guyannensis*. PS1, *P. guyannensis* (2n = 46); PG, *P. gardneri* (2n = 40); PS2, *P. guyannensis* (2n = 38); PE, *P. echinothrix* (2n = 32); PL, *P. longicaudatus* (2n = 28); PC, *P. cuvieri* (2n = 28). **(A)** L1 retroelement hybridization in PS1. **(B)** L1 retroelement hybridization in PG. **(C)** L1 retroelement hybridization in PS2. **(D)** L1 retroelement hybridization in PE. **(E)** L1 retroelement hybridization in PL. **(F)** L1 retroelement hybridization in PC. 10.0 µm.

**Figure S2.**
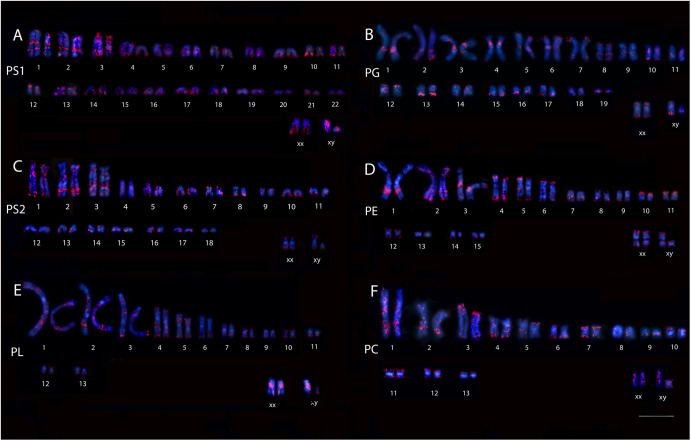
Karyotype of the five species of *Proechimys*, male and female, including two cytotypes of *P. guyannensis*. PS1, *P. guyannensis* (2n = 46); PG, *P. gardneri* (2n = 40); PS2, *P. guyannensis* (2n = 38); PE, *P. echinothrix* (2n = 32); PL, *P. longicaudatus* (2n = 28); PC, *P. cuvieri* (2n = 28). **(A)** SINE-B1 retroelement hybridization in PS1. **(B)** SINE-B1 retroelement hybridization in PG. **(C)** SINE-B1 retroelement hybridization in PS2. **(D)** SINE-B1 retroelement hybridization in PE. **(E)** SINE-B1 retroelement hybridization in PL. **(F)** SINE-B1 retroelement hybridization in PC. 10.0 µm.

The L1 and B1 consensus submitted to Repbase database (Bao et al, 2015) provided a non-LTR retrotransposon sequence with 509 bp and 90% identity with *Chinchilla lanigera* coincident with structural variants L1-3_Clan (Kojima, 2019), and with 149 bp and 84% identity with *Cavia porcellus* (Jurka, 2010) coincident with structural variants ID-B1_Cpo, respectively.

Although dispersed in chromosomal pairs, the L1 pattern remained conserved when the genomes of *Proechimys* species were compared (Fig 1). For example, the accumulation of L1 in chromosomal pair 1 of PC is similar to the regions corresponding to the pairs 10 + 18 + 15 + 4 of PS1, and to the pairs 6 + 8 of PS2, demonstrating possible rearrangements in the chromosomes of these species as indicated by the arrows (Fig 1 and Table 1).

**Table 1.** Probable rearrangements in chromosomes of *Proechimys* spp. from the distribution of the retroelement LINE-1 as shown in Fig 1.

PS1 (2n = 46)	PG (2n = 40)	PS2 (2n = 38)	PE (2n = 32)	PL (2n = 28)	PC (2n = 28)
10a + 18a + 15a + 4a		6a + 8sm			1m
7a + 9a + 14a	12m + 6sm + 18a +14a + 16a	11a + 10a + 5a + 16a	8m + 10m+ 4sm	1m	11m + 10a+ 6sm +12m
1a	3sm + 1a		6st + 5sm	9a + 10m + 6m	5m
3a	9a + 13m	1st	12m + 3st		7m + 3a
17a + 12m + 13m				11m + 12m + 3a	2st
2a	5m + 8m + 10a	14a + 7m + 12a			
20a + 16a	11a + 2a		2sm	2st	9sm + 8a
19a + 6a		2st	1sm	5m	
8a		3st			
5a				4sm	4m
	4m			8sm	
	7a	4sm	7a		
21a	17a	9m	11m	7m	
11sm	15m	13a	9m		
		17a	14m + 15m		
22a		15a			
	19a	18m		13a	
			13m		13m

PS1, *P. guyannensis* (2n = 46); PG, *P. gardneri* (2n = 40); PS2, *P. guyannensis* (2n = 38); PE, *P. echinothrix* (2n = 32); PL, *P. longicaudatus* (2n = 28); PC, *P. cuvieri* (2n = 28); m, metacentric; sm, submetacentric; st, subtelocentric; a, acrocentric.

The same pattern can be observed between the chromosome pairs of the different species. In at least one group of chromosomes, total correspondence between the species was observed: pairs 7 + 9 + 14 of PS1, 1 of PL, 12 + 6 + 18 + 14+16 of PG, 8 + 10 + 4 of PE, 11 + 10+5 + 16 of PS2 and 11 + 10+6 + 12 of PC, including the two cytotypes of *P. guyannensis* (Fig 1 and Table 1). L1 was not present in chromosome pairs 17 of PS2 and 14 + 15 of PE. The homeology in each group of *Proechimys* species comprised the centromeric and/or pericentromeric regions of virtually all the chromosomes that were compared (Fig 1).

L1 was dispersed in the X chromosomes, usually labeling the centromeric/pericentromeric and proximal regions, with the exception of PG. The distribution of L1 in the Y chromosomes was variable, encompassing one or both chromosome arms. It was present in both chromosome arms in PS1, PE, and PC, on the Yp in PL, on the Yq of PG and PS2, and on the pericentromeric regions of PS1, PL, and PC. Conspicuous labeling was observed in the interstitial and/or centromeric region of PS1, PS2, and PC and terminal region of PE.

Physical mapping of B1 in the karyotype of the five analyzed species of *Proechimys* showed a pattern with preponderance to compartmentalization and, as for L1, established a complete homeology in most chromosomal arms (Fig 2).

The B1 chromosome distribution pattern was conserved among species, especially in terminal regions, such as in chromosome pairs 2 of PL, PC and PS1, 3 of PS2, and 5 of PG, demonstrating possible rearrangements in the chromosomes of these species as indicated by the arrows (Fig 2 and Table 2).

**Table 2.** Probable rearrangements in chromosomes of *Proechimys* spp. from the distribution of retroelement SINE B1 as shown in Fig 2.

PS1 (2n = 46)	PG (2n = 40)	PS2 (2n = 38	PE (2n = 32)	PL (2n = 28)	PC (2n = 28)
				1m	1m
2a	5m	3st		2st	2st
21a + 6a + 10a		1st	2sm	3a	
1a	3sm	2st			
	1a		1sm		
3a	13m + 14a			7m + 13a	3a
4a	2a				
	18a + 15m			4sm	4m
7a	6sm		3st		13m + 8a + 10a
15a + 19a	11a + 19a	9m		5m	5m
	4m	4sm			
20a + 17a	10a	17a + 10a	4sm	6m	
	7a		5sm		
13m + 12m	8m	14a + 8sm	6st	10m + 8sm	12m + 7m
	9a		9m		
5a		5a		9a	
11sm			10m		
	12m	7m	8m		
14a	17a				6sm
8a		11a			
18a		6a	7a		
16a		12a	12m		11m
		18m	11m		
	16a	13a		12m	
9a		15a	15m	11m	
			13m		9sm
22a		16a	14m		

PS1, *P. guyannensis* (2n = 46); PG, *P. gardneri* (2n = 40); PS2, *P. guyannensis* (2n = 38); PE, *P. echinothrix* (2n = 32); PL, *P. longicaudatus* (2n = 28); PC, *P. cuvieri* (2n = 28); m, metacentric; sm, submetacentric; st, subtelocentric; a, acrocentric.

As for L1, the signal of B1 had full equivalence between species in at least one chromosomal group, including the two cytotypes of *P. guyannensis*, which comprises the chromosome pairs 12 + 7 of PC, 6 of PE, 8 of PG, 10 + 8 of PL, 14 + 8 of PS2 and 13 + 12 of PS1 (Fig 2 and Table 2). The B1 signal was present in the centromeric/pericentromeric region of all chromosomes of each group of *Proechimys* species compared (Fig 2).

In the X chromosomes, B1 distribution varied among the five analyzed species: it was seen in both arms in PG, PS2, PE, PL, and PC, on the Xq of PS1. B1 was mapped on in both arms in PS1, PS2, PE, PL, and PC, Yq of PG. B1 signals on the sex chromosomes were characterized by the presence of conspicuous blocks in the X chromosome, and involved the centromeric regions of PG, PE, and PL. On the Y chromosome, the signals occurred in the interstitial region of PS1, PG, and PC, and terminal of PS2, PE, and PL, and included the centromeric regions of PS1, PS2, and pericentromeric areas of PC (Fig 2).

Our data show a predominance of conspicuous blocks of B1 over L1, and a signal correlation of these retroelements on sex chromosomes in virtually all *Proechimys* species analyzed (Figs 1 and 2).

**Figure 3.**
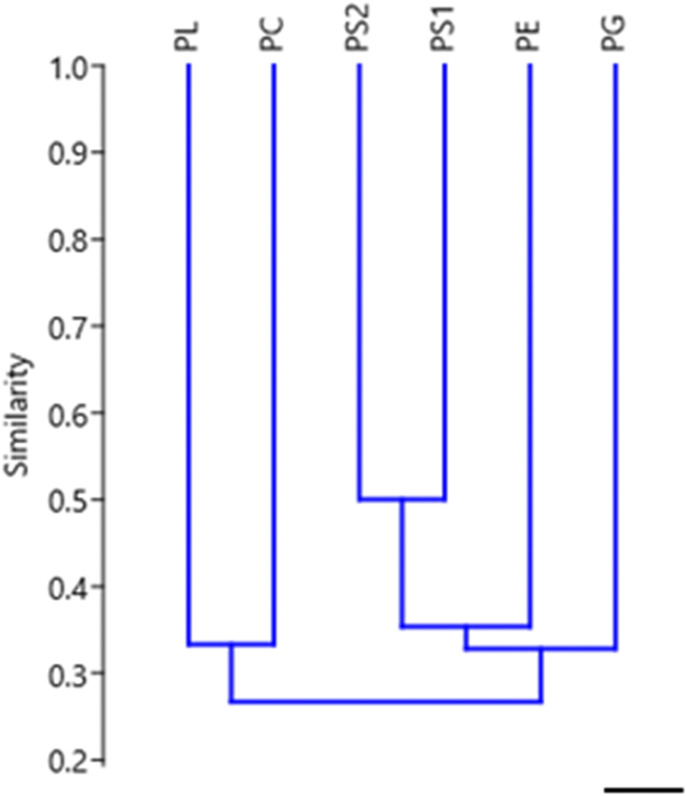
Cladogram obtained after the analysis in PAST 4.3 of the species. PS1, *P. guyannensis* (2n = 46); PG, *P. gardneri* (2n = 40); PS2, *P. guyannensis* (2n = 38); PE, *P. echinothrix* (2n = 32); PL, *P. longicaudatus* (2n = 28); PC, *P. cuvieri* (2n = 28); using the possible chromosomal rearrangements generated by the retroelements L1 and B1 as single characters (Tables S1 and S2). 10.0 µm. Source data are available online for this figure.

The probable structural rearrangements from the distribution of L1 and B1 retroelements allowed the grouping of the examined *Proechimys* species according to similarity levels (Fig 3 and Table 3).

**Table 3.** Jaccard Similarity index obtained in the comparisons among species.

	PS1	PG	PS2	PE	PL	PC
PS1	1	—	—	—	—	—
PG	0.375	1				
PS2	0.564103	0.35	1			
PE	0.325	0.314286	0.444444	1		
PL	0.472222	0.314286	0.368421	0.257143	1	
PC	0.428571	0.30303	0.225	0.322581	0.464286	1

**Figure 4.**
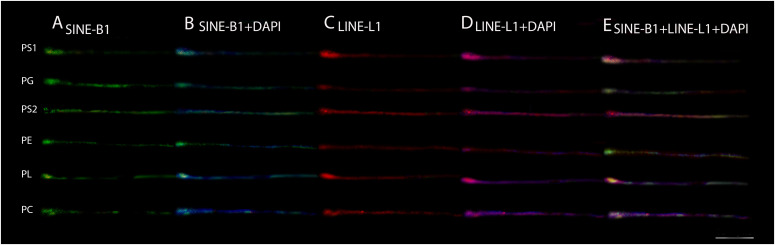
Fiber-FISH showing adjacent and overlapping LINE–L1 (red) and SINE-B1 (green) cluster in *Proechimys* spp. PS1, *P. guyannensis* (2n = 46); PG, *P. gardneri* (2n = 40); PS2, *P. guyannensis* (2n = 38); PE, *P. echinothrix* (2n = 32); PL, *P. longicaudatus* (2n = 28); PC, *P. cuvieri* (2n = 28). **(A, B, C, D, E)** SINE-B1, (B) SINE-B1 + DAPI, (C) LINE-L1, (D) LINE -L1 + DAPI, (E) SINE-B1 + LINE-L1+ DAPI. 10.0 µm. Source data are available online for this figure.

The cladogram generated from the distribution of L1 and B1 retroelements in the different karyotypes of *Proechimys* revealed two major groups composed of four subgroups with different degrees of similarity: (1) PL and PC, (2) PS1 and PS2, (3) PE, and (4) PG (Fig 3 and Table 3) (Tables S1 and S2). Fiber-FISH analysis allowed the determination of signals from the L1 and B1 retroelements in the genome of *Proechimys* ssp. (Fig 4).

Table S1 Basic data matrix for LINE-1. Number of characters: 19. Number of informative characters: 19.

Table S2 Basic data matrix for SINE-B1. Number of characters: 27. Number of informative characters: 27.

The pattern of L1 and B1 observed in the genome of *Proechimys* species, through fiber-FISH, revealed an adjacent and overlapping distribution in a differentiated way among the species. PS1 and PS2 with L1 overlap along the fiber and formation of B1 blocks preferably in the end tip, PL and PC with overlap at the beginning, alternating L1 with B1 blocks along the fiber, PG with B1 blocks in the middle and L1 dispersed preferably in the end tip, PE with L1 dispersed in the middle, with some overlap and B1 in the terminal regions (Fig 4).

**Figure 5.**
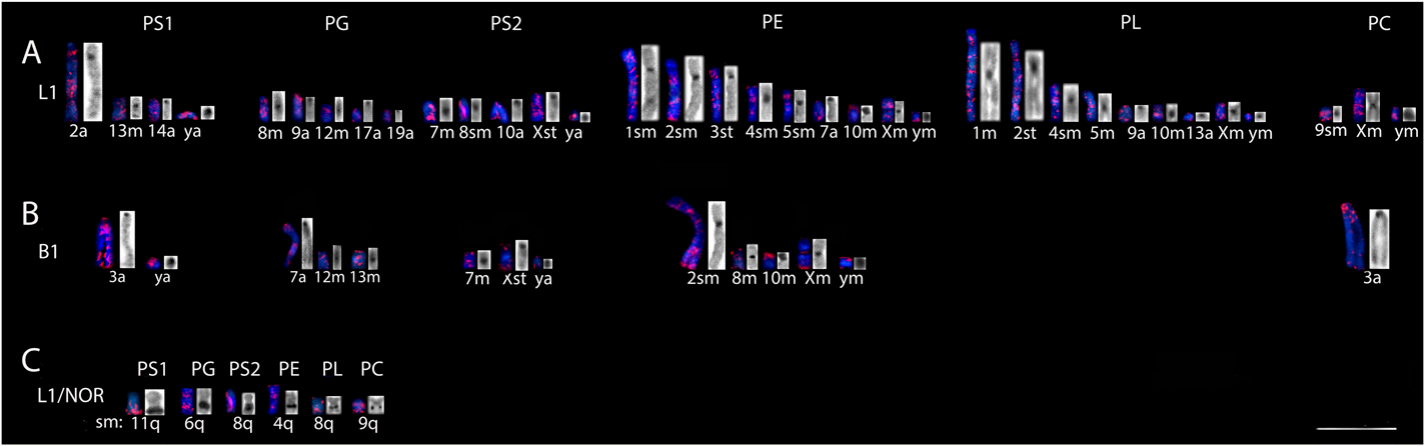
Hybridization correspondence among LINE-1, SINE-B1, nucleolus organizer regions, and constitutive heterochromatin (CH) in chromosomes of *Proechimys*. PS1, *P. guyannensis* (2n = 46); PG, *P. gardneri* (2n = 40); PS2, *P. guyannensis* (2n = 38); PE, *P. echinothrix* (2n = 32); PL, *P. longicaudatus* (2n = 28); PC, *P. cuvieri* (2n = 28). **(A)** LINE-1 on the left and CH on the right. **(B)** SINE-B1 on the left and CH on the right. **(C)** LINE-1 on the left and Ag-NOR on the right. m, metacentric; sm, submetacentric; st, subtelocentric; a, acrocentric; q, long arms. 10.0 µm. Source data are available online for this figure.

Comparisons of NOR sites with L1 and B1 retroelements demonstrated coincidence with L1, but not with B1 signals on the long arm of submetacentric chromosomes of five examined *Proechimys* species. And both retroelements marked in regions rich in constitutive heterochromatin (CH) of some chromosomes (Fig 5 and Table 4).

**Table 4.** Hybridization correspondence among LINE-1, SINE-B1, NORs, and constitutive heterochromatin (CH) in chromosomes of *Proechimys* as shown in Fig 4.

*Proechimys *spp.	PS1 (2n = 46)	PG (2n = 40)	PS2 (2n = 38)	PE (2n = 32)	PL (2n = 28)	PC (2n = 28)
L1 signal corresponding to constitutive heterochromatin	2a/13m/14a/Ya	8m/9a/12m/17a/19a	7m, 8sm, 10a, Xst, Ya	1sm, 2sm, 3st, 4sm, 5sm, 7a, 10m, Xm, Ym	1m, 2st, 4sm, 5m, 9a, 10m, 13a, Xm, Ym	9m, Xm, Ym
B1 signal corresponding to constitutive heterochromatin	3a, Ya	7a, 12m, 13m	7m, Xst, Ya	2sm, 8m, 10m, Xm, Ym	-	3a
NORs sites coincident with the L1 signal	11sm	6sm	8sm	4sm	8sm	9sm

PS1, *P. guyannensis* (2n = 46); PG, *P. gardneri* (2n = 40); PS2, *P. guyannensis* (2n = 38); PE, *P. echinothrix* (2n = 32); PL, *P. longicaudatus* (2n = 28); PC, *P. cuvieri* (2n = 28);m, metacentric; sm, submetacentric; st, subtelocentric; a, acrocentric.

Sites rich in CH were coincident with centromeric and/or pericentromeric regions of some chromosomes with L1 and B1 signals in *Proechimys* spp. (Fig 5A and B and Table 4).

Several unique characteristics were observed in some species: in *P. guyannensis* (2n = 46), the L1 signal on chromosome 2a was coincident with CH in a region close to the centromere; in *P. echinothrix*, blocks of L1 and B1 coincided with the fully heterochromatic short arm of metacentric 10; and in *P. longicaudatus*, there was no B1 labeling that coincided with heterochromatin. The totally heterochromatic PS1 and PE Y chromosome showed blocks of L1 and B1, and in PS2 and PC there were also strong labeling with L1 (Fig 5A and B).

## Discussion

The physical mapping of L1 and B1 in *Proechimys* species followed the pattern proposed by Charlesworth et al (1994), with interspersed signals in most chromosomes, as was described in cricetid rodents *Microtus agrestis* and *Microtus rossiaemeridionalis* (Neitzel et al, 1998, 2002). The arrangement of these retroelements with a conserved pattern in five species of *Proechimys*, including two cytotypes of *P. guyannensis*, allowed a comparison among corresponding chromosomes, sometimes involving the centromeric regions (Figs 1 and 2 and Tables 1 and 2). The similarities observed among corresponding chromosomes of these five species of *Proechimys* suggest signals of L1 and B1 in pericentromeric regions, and indicate rearrangements during karyotype diversification, as proposed by Paço et al (2015) and Araújo et al (2017).

The distribution of L1 and B1 retroelements in the different karyotypes presented in this study revealed chromosomal homeologies in *Proechimys* comprising four groups: (1) PL and PC, (2) PS1 and PS2, (3) PG, and (4) PE. These data reflect some of the taxonomic relationships proposed by Patton et al (2015) for *Proechimys*: PS1 and PS2, corresponding to the two cytotypes of *P. guyannensis* (Guyenne spiny rat), were grouped together, as were PL and PC, corresponding to *P. longicaudatus* (Long-tailed spiny rat) and *P. cuvieri* (*Cuvier*’s spiny rat), both of which belong to the *longicaudatus* group according to Patton (1987) and Patton et al (2015). Considering the structural rearrangements proposed, it is plausible that L1 and B1 are involved in the karyotype reorganization of this taxa (Figs 1 and 2). The degree of similarity between the formed clusters showed that retroelements L1 and B1 can behave as derived characters shared in *Proechimys* (Fig 3). The distribution patterns of the TE sequences demonstrate similarities between the species, which infers that these retroelements may be involved in fusion/fission-type rearrangements in the chromosomes of the groups proposed (Figs 1, 2, and 3). These data provide insights into the evolutionary relationships between taxa, revealing cryptic species and contributing to the fine-tuning of phylogenetic trees. Studies of TE sequences in *Proechimys* involving a larger number of species seem promising to clarify the phylogenetic relationships of species-rich genera such as *Proechimys* (Boyle et al, 1990; Ricci et al, 2018; D’Elía et al, 2019).

The distribution pattern of L1 and B1 in the chromosomal fibers of *Proechimys* species demonstrates that these retroelements were organized in different locations in the genome of each species in an interspersed and/or colocalized way, comprising four groups, as shown in the cladogram, because of the signal similarity between the fibers (Figs 3 and 4). The pattern of the L1 and B1 signal in the chromosomal fiber of *Proechimys* confirms the data provided by the possible rearrangements, as it demonstrates that these retroelements remained in the genome of different species organized differently (Figs 1, 2, 3, and 4). Fiber-fish has been shown to be a tool to accurately map repetitive sequences, making it possible to assess whether they are overlapping or adjacent in the genome as in this study (Wang et al, 2013; Souza et al, 2021).

The hybridization signals of retroelements in the sex chromosomes of *Proechimys* species followed the same pattern as in the autosomes, in which L1 is accumulated mainly in interstitial regions, whereas B1 is centromeric/pericentromeric (Figs 1 and 2), as reported by Chen and Manuelidis (1989). The L1 labeling in these chromosomes presented similar interspersed and non-random patterns to those obtained in the rodent species *Mus musculus* and *Peromyscus maniculatus* (Wichman et al, 1992; Baker & Kass, 1994). However, the pattern of B1 labeling differed from that reported for *M. musculus* and three species of the Akodontini rodent tribe (Boyle et al, 1990; Araújo et al, 2017), which showed no preference for the sex chromosomes. Sex chromosome analyses have demonstrated a significant accumulation of specific TEs at particular sites as reported by Erlandsson et al (2000) and Dong et al (2017).

Regarding the accumulation of L1 and B1 on Y chromosomes, as observed in PS1/PS2/PC and PS1, respectively (Figs 1 and 2), may be related to epigenetic mechanisms. Studies in humans, chimpanzees, and mice indicate that this pattern, suggesting an involvement of TEs, L1 and B1, in Y chromosome divergence in mammals (Erlandsson et al, 2000; Simonti et al, 2017; Rojas-Ríos & Simonelig, 2018; Tang et al, 2018).

The high accumulation of L1 in the interstitial regions of X chromosomes of the five *Proechimys* species examined, including the two cytotypes of *P. guyannensis*, supports Lyon (1998) hypothesis that the L1 retroelement is associated with X chromosome inactivation. In studies of embryonic stem cells in mice, Chow and Heard (2009) and Chow et al (2010) have shown that specific regions composed by clusters of LINEs may be involved in the recruitment of Xist RNAs in the process of X chromosome silencing.

The terminal and pericentromeric regions of some chromosomes of *Proechimys* examined here were labeled with retroelement probes. Some centromeric regions had strong labeling coincident with CH: L1 in PS1 (Ya), PG (9a, 12m), PS2 (7m, 8sm, and Ya), PE (10m, 4sm), PL (10m) and PC (9sm, Ym), and B1 in PS1 (Ya), PG (13m), PE (10m, Ym), and PC (3a) (Fig 5A and B). Our results agree with those presented by Schueler et al (2001), in which pericentromeric sites in humans were shown to have large blocks of LINE and SINE.

In *Proechimys* sex chromosomes, L1 and B1 retroelements seemed to accumulate in the centromeric/pericentromeric regions (Figs 1 and 2), a characteristic also reported in other rodents such as *M. agrestis* and *M. rossiaemeridionalis* (Neitzel et al, 1998, 2002). The alternation of L1 and/or B1 at heterochromatic centromeric sites on the sex chromosomes of several *Proechimys* species (Fig 5A and B) may be associated with competition among TE copies present in the same genome, as suggested by Hua-Van et al (2011).

The particularity observed on chromosome 2a of PS1 that presented L1 blocks and CH close to the centromere, and the L1/B1 blocks in PE coincident with the fully heterochromatic short arm of metacentric pair 10 (Fig 5A and B) may be associated with the plasticity of the genome in heterochromatin remodeling. Nishibuchi and Déjardin (2017) suggested that derepression of TEs may result in the redistribution and reorganization of heterochromatin, which normally restricts the activity of these elements. The presence of TEs in centromeric regions, associated or not with heterochromatin, has been suggested to be part of the dynamics and architecture of the mammalian genomes. In this sense, some studies have shown that these sequences are inserted in the centromeric domains, in order to be used for the recruitment of histones and formation of new centromeres (Schueler et al, 2001; Kuznetsova et al, 2006; Meyer et al, 2016).

In *Proechimys*, specific characteristics of these retroelements stand out, such as the formation of conspicuous blocks, especially in the sex chromosomes (Figs 1 and 2). It is possible that this accumulation of L1 and/or B1 sequences at specific sites in the genome of these rodents occurred in a non-random way, this may be an evolutionary strategy such that TEs reduce their deleterious impact on the genome (McGurk et al, 2021). Rebollo et al (2011) and Chuong et al (2017) have suggested that the groupings of TEs in certain regions could substantially change gene regulation or even influence simultaneous expression of multiple genes in specific contexts, despite deleterious effects and repression of gene activity. The interactions among these sequences and specific genome sites is a striking feature, and is considered to reflect the functionality of TEs because they may play an important role in genomic plasticity and adaptive behavior (Bourque et al, 2018; Razali et al, 2019; Gonçalves et al, 2020).

The localization of L1 and B1 retroelements in the terminal region of chromosomes of several *Proechimys* species, including sex chromosomes (Figs 1 and 2), may be related to their association with the subtelomeric heterochromatin and their role on the regulation of telomere elongation, as seen in other mammals (Gonzalo et al, 2006; Slotkin & Martienssen, 2007).

TEs have been previously found close to NORs, as detected in this study, because this site coincided with L1 signal, but not with B1, in a NOR-bearing submetacentric chromosome in all species (Fig 5C). A similar pattern was observed with the mariner-like element (MLE), that mapped close to the heterochromatin associated with NORs in the water snail *Theodoxus fluviatilis*, and in the fish *Gobius niger* (Mandrioli et al, 2001; Mandrioli & Manicardi, 2001). The presence of TEs in regulatory regions of the genome occurs so that these retroelements contributed to these regions, a mechanism known as exaptation (Chuong, 2013; Lynch et al, 2015).

Simonti et al (2017) demonstrated in human and mouse cells that ancient elements are more likely to overlap with a regulation enhancer in the genome. It is also possible that the sequences of L1 and not B1, inserted close to the matrix of rRNA genes in the chromosomes of *Proechimys* species, may have been co-opted to potentiate their regulatory effect. This hypothesis may be evaluated taking into account the results presented here, because B1 is younger than L1, and arose ca. 65 MYA after rodent divergence (Kramerov & Vassetzky, 2005; Yang et al, 2019).

The retroelements L1 and B1 seem to be related to the karyotype diversification of *Proechimys* species analyzed in this study, in accordance with Dobigny et al (2004a, 2004b), who demonstrated that L1 amplification accompanied karyotype evolution in species of *Taterillus*.

Karyotype diversification in *Proechimys* may have occurred through fixation of chromosomal rearrangements and, when associated with pericentromeric or subtelomeric regions, may represent a chromosomal breakdown hotspot with recombination between homologous and non-homologous chromosomes. Similarities in L1 and B1 distribution among chromosomes of *Proechimys* species seems to derive from a trend towards chromosomal rearrangements, evidenced by the different organization of these retroelements in the chromosomal fiber, which maintain a diverse karyotype pattern in their evolutionary relationships.

Karyotype diversity in the genus *Proechimys* has been repeatedly cited in the literature, but the governing principles of this variation remain obscure. The present study expands our understanding of the genome of this genus by mapping two TEs, allowing to assess chromosomal homologies among the taxa and deduce characters in phylogenetic investigations. The distribution of L1 and B1 may represent a source for karyotypic diversity, which may be revealed in future studies if they are shown to be related to rearrangements breakpoints.

## Materials and Methods

The five species of *Proechimys* analyzed are listed in Table 5. They were collected in the Brazilian Amazon from 2005 to 2015 with authorization under permits 02005.000642/03-11 (IBAMA/MMA), 02000.002336/2003-93 (IBAMA/MMA), 02005.002672/04 (IBAMA/MMA), 37585-5 (SISBIO/MMA), 37592-4 (SISBIO/MMA), 10985 (SISBIO/MMA). The skins and skulls were deposited at the Mammal Collection of the Instituto Nacional de Pesquisas da Amazônia (INPA). Cytogenetic analyses were performed on chromosome preparations obtained from bone marrow cells, using colchicine at a concentration of 0.0125%, 1 ml for each 100 g of animal mass (Ford & Hamerton, 1956). The cell suspensions and tissue samples for molecular cytogenetic studies are deposited in the LGA at INPA (Silva et al, 2012; Eler et al, 2012, 2020).

**Table 5.** Species of *Proechimys* analyzed.

Species	Sample location	Coordinates	Collection	2n/FN
*P. guyannensis* ^a^	Serrinha Island, Balbina hydroelectric dam, Uatumã River, Amazonas	01°52’ S, 59°25’ W	INPA-CEF12	46/50^b^
			INPA-CEF14	
*P. guyannensis* ^a^	Monte Dourado, Almeirim, Pará	00°49’ S, 52°39’ W	INPA5053	38/52^c^
			INPA5054	
*P. gardneri*	Left bank of the Madeira River, Bela Vista Community, Amazonas	05°14’ S, 60°42’ W	INPA5383	40/54^c^
			INPA5390	
*P. echinothrix*	Canutama Extractive Reserve, Purus River, Amazonas	06°34’ S, 64°33’ W	INPA7319	32/58^c^
			INPA7345	
*P. longicaudatus*	Left bank of the Aripuanã River, Amazonas	06°17’ S, 60°23’ W	INPA5414	28/46^c^
			INPA5401	
*P. cuvieri*	REMAM Forest (Isaac Sabbá Refinery), Manaus, Amazonas	00°70’ S, 52°67’ W	INPA-EE251	28/46^b^
	Bituba, Monte Dourado, Almeirim Pará	01°11’ S, 52°38’ W	INPA5050	

2n, diploid number; FN, fundamental number; INPA, Instituto Nacional de Pesquisas da Amazônia.

a Different cytotypes for *P. guyannensis* 2n = 46^1^ and 2n = 38^2^.

b Silva et al(2012).

c Eler et al(2012).

The retroelements L1 and B1 were obtained using polymerase chain reaction (PCR). The following primer sets were used: L1-F (5′-AAGAATTCCGCAGGATACAAGATCAACTCA-3′) and L1-R (5′-AAGGATCCCAATTCGATTCCATTGGT-3′) (Grahn et al, 2005), B1-F (5′-GCCGGGCGTGGTGGCG-3′) and B1-R (5′-TTGGTTTTTCGAGACAGGGTTTCT-3′) (Rinehart et al, 2005). The PCR products were purified with the SV Gel Wizard kit and PCR cleaning system (Promega), and cloned into the pGEM-t Easy Vector kit (Promega). The recombinant plasmids were sequenced on the ABI3130 platform (Myleus Biotechnology), and the sequences were submitted to the Repbase database for consensus (https://www.girinst.org/censor/) and are available in GenBank with the numbers https://www.ncbi.nlm.nih.gov/nuccore/MW027222 and https://www.ncbi.nlm.nih.gov/nuccore/MW027223. Sequenced plasmids were tagged by nick translation with digoxigenin-11-dUTP (DIG-Nick translation Mix; Roche Applied Science), according to the manufacturer’s instructions, and used as probes for FISH. The slides containing the target chromosomes were initially treated with pepsin for 10 min at 37°C and washed in distilled water. Chromosomal denaturation was performed in 70% formamide in 2× SSC at 70°C for 2 min followed by dehydration in ethanol. The hybridization mix consisted of 200 ng of digoxigenin-labeled probe and hybridizations in hybridization buffer (50% formamide/20× SSC/50% dextran sulfate) were performed at 42°C over night, with 77% stringency. After post-hybridization washings in 2×SSC/PBST at 37°C and immunodetection with rhodamine-conjugated antidigoxigenin, the metaphases were counterstained with DAPI (0.8 ng/μl) in antifade reagent (SlowFade; Invitrogen).

For fiber-FISH experiments, the protocol by (Barros et al, 2011) was used, with some modifications. The material in cell suspension was placed on glass slides moistened in water at 65°C, washed in saline solution (1× PBS/1 m), followed by successive baths in an alcoholic series. 800 μl of 0.5 M NaOH solution (diluted in 30% ethanol) were added and the chromosomal fibers were elongated, followed by the immediate application of 500 μl of 100% ethanol. Then FISH was performed with LINE-L1 and SINE-B1 probes.

The results obtained using FISH for the retroelements L1 and B1 were compared to the CBG-banding patterns and the silver-staining of the nucleolus organizer regions (Ag-NORs) described by Eler et al (2012, 2020) and Silva et al (2012) because the same specimens were used in all those studies.

The probable structural rearrangements in the chromosomes of *Proechimys* species from the distribution of L1 and B1 retroelements were coded as binary characters, and used in a cluster analysis using a dendrogram performed with PAST 4.3 software. The comparison of similarity among species was performed using the Jaccard similarity index shown in Table 3 (Hammer et al, 2001). A data matrix was established based on the presence or absence of chromosomal homology characters as suggested by Dobigny et al (2004a, 2004b) (Tables S1 and S2).

## Data Availability

All raw and processed sequencing data demonstrated in this study have been submitted to the NCBI GenBank (https://www.ncbi.nlm.nih.gov/genbank) under accession number https://www.ncbi.nlm.nih.gov/nuccore/MW027222 and https://www.ncbi.nlm.nih.gov/nuccore/MW027223.

## Supplementary Material

Reviewer comments
