# Supplementary figures and images for: LINE-1 and SINE-B1 mapping and genome diversification in Proechimys species (Rodentia: Echimyidae)
Source: Life Sci Alliance. 2022 Mar 18;5(6):e202101104. doi: 10.26508/lsa.202101104 (PMC8932440; doi:10.26508/lsa.202101104)

# LINE-L1

PS1

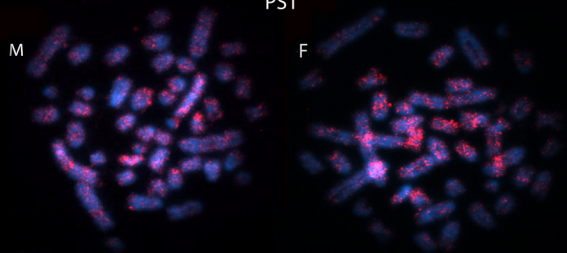

PG

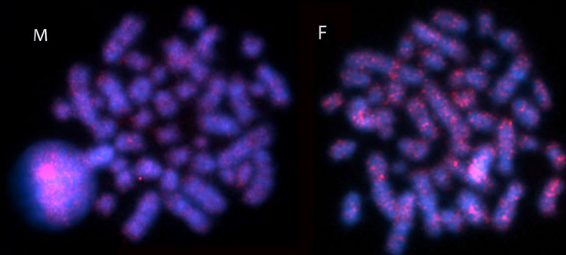

PS2

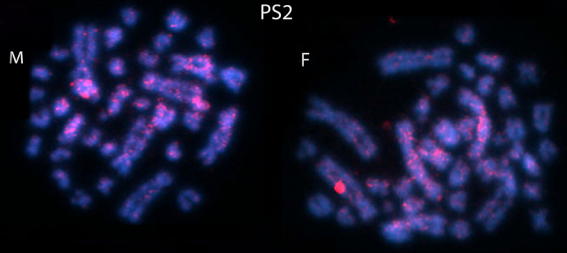

PE

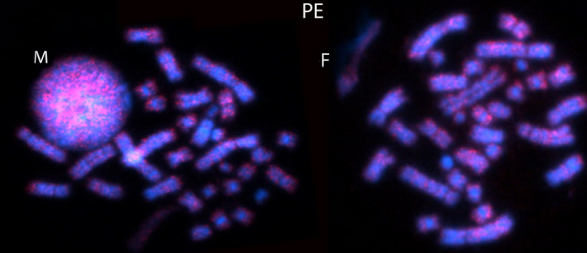

PL

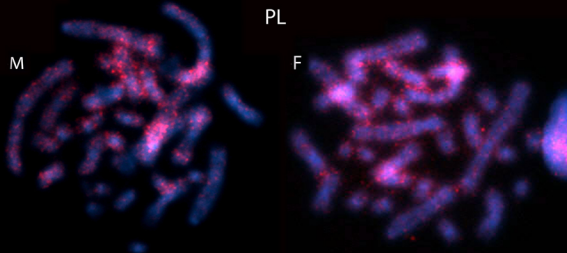

PC

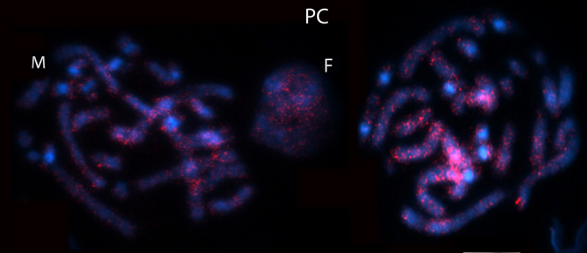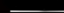

Supplement: Supplementary file 1 [file LSA-2021-01104_SdataF1.pdf]

# SINE-B1

PS1

M

F

PG

F

PS2

M

F

PE

F

PL

M

F

PC

F

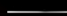

Supplement: Supplementary file 2 [file LSA-2021-01104_SdataF2.pdf]

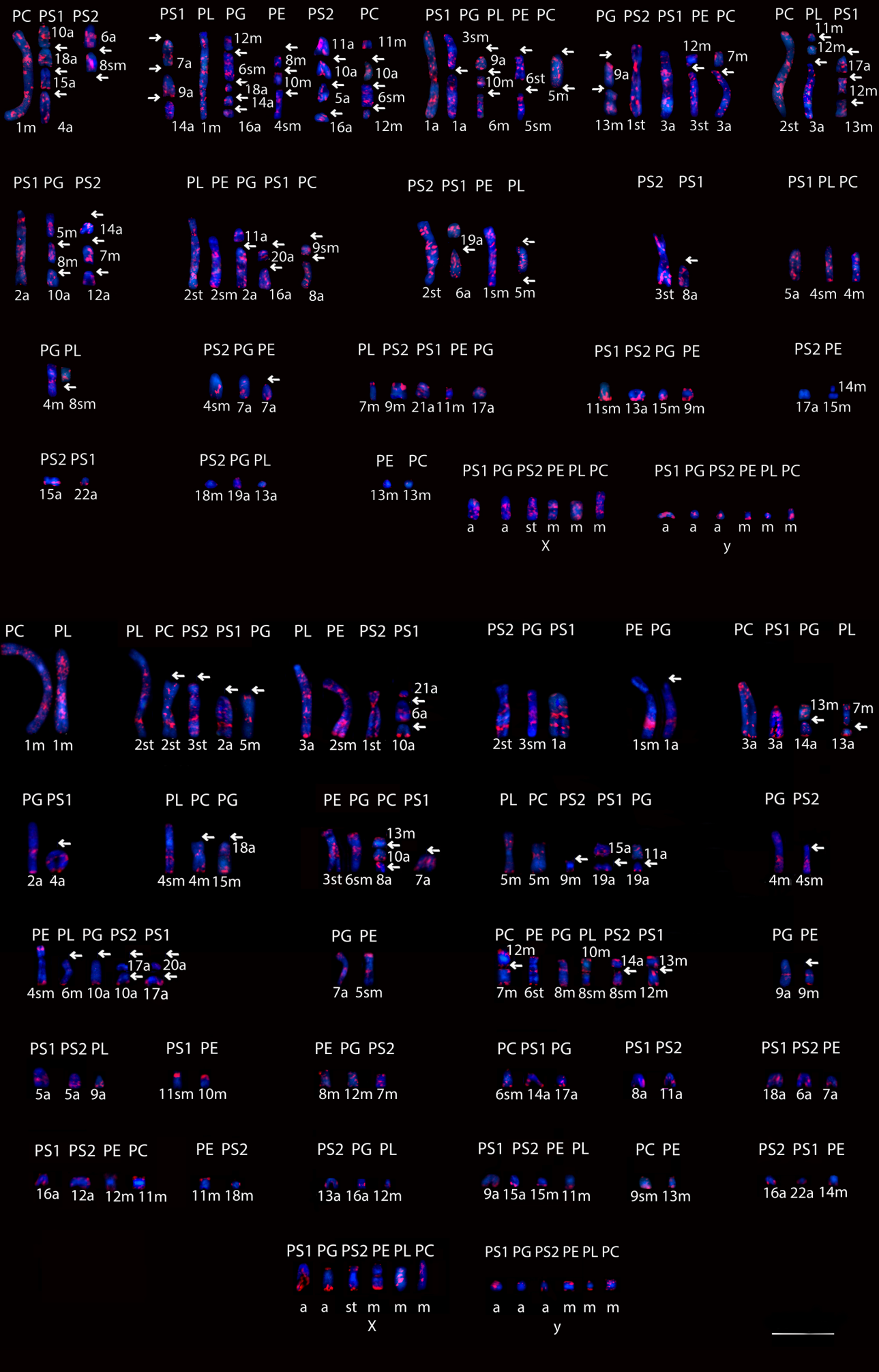

Supplement: Supplementary file 3 [file LSA-2021-01104_SdataF3.pdf]

# Fiber-FISH

PS1

PG

PS2

PE

PL

PC

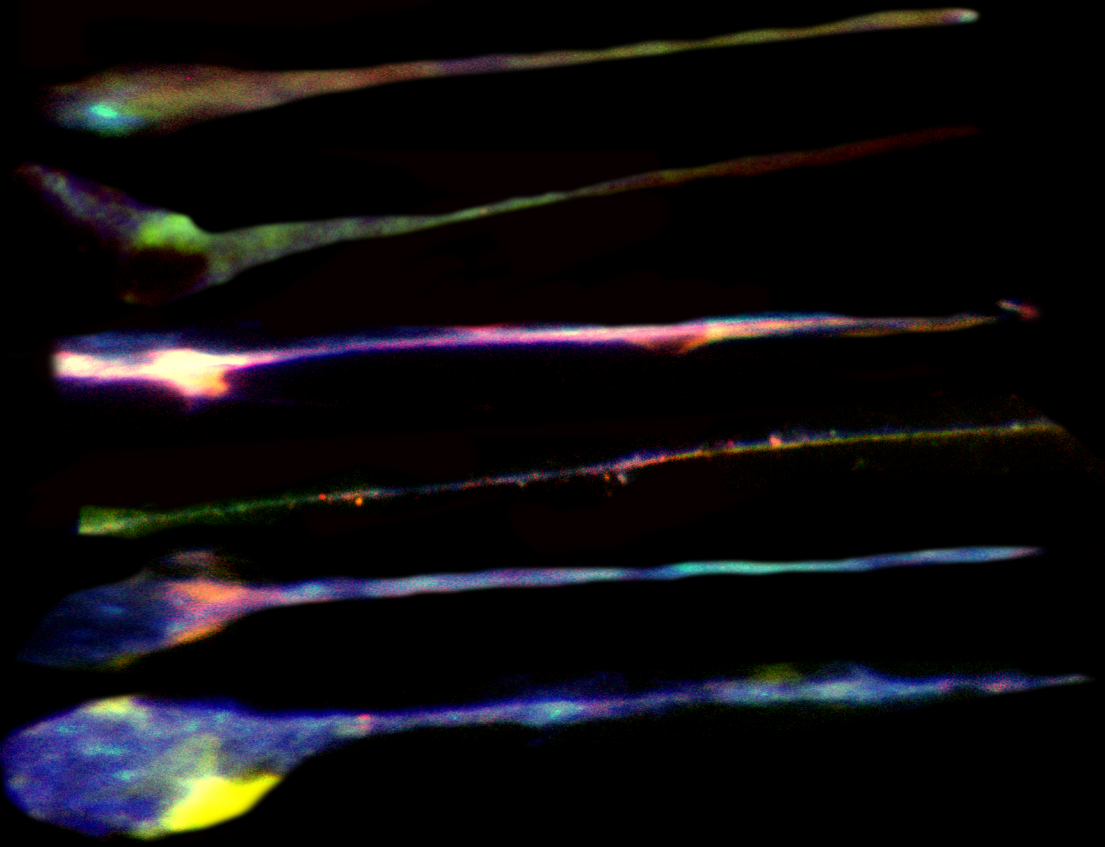

Supplement: Supplementary file 6 [file LSA-2021-01104_SdataF4.pdf]

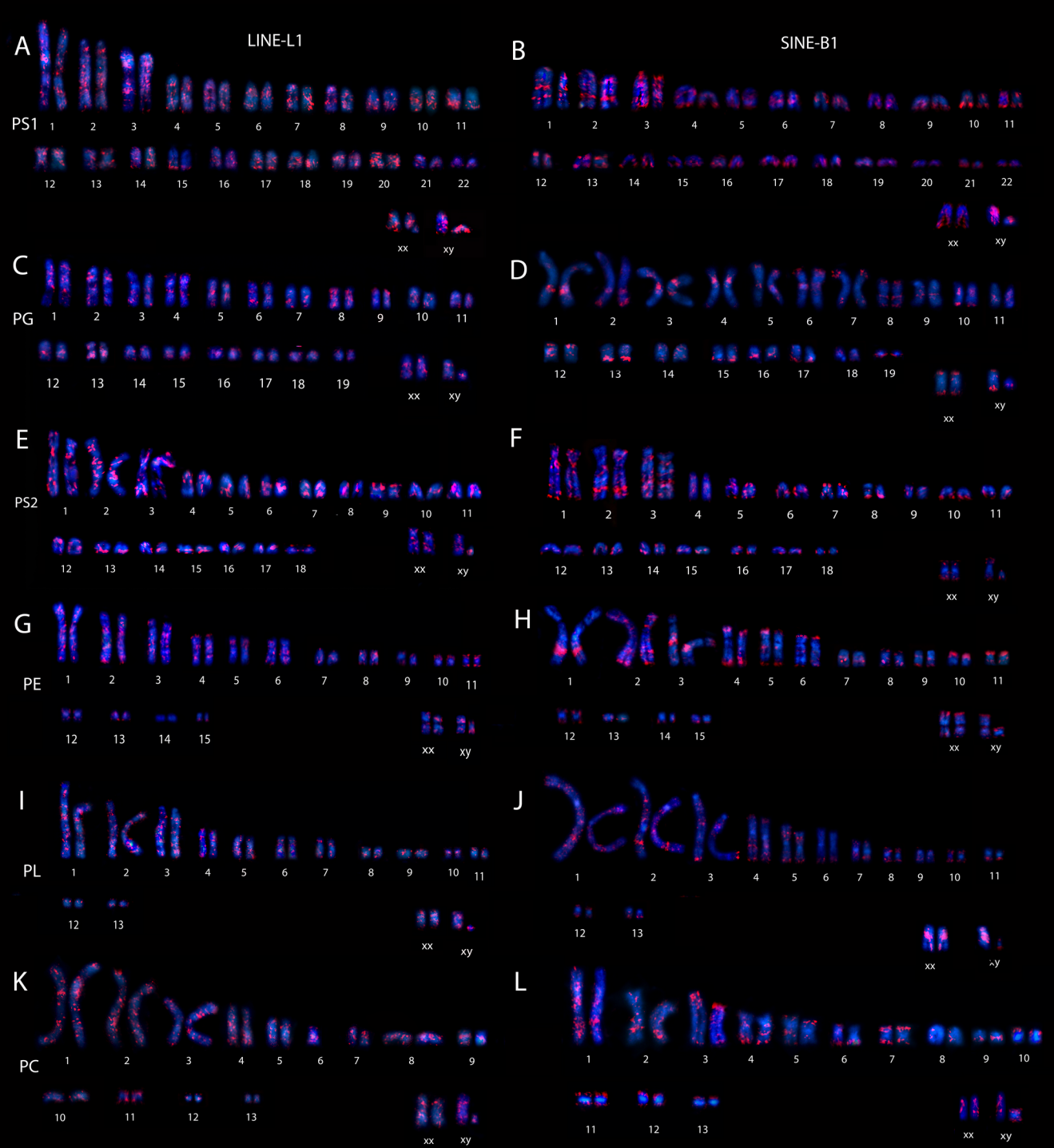

Supplement: Supplementary file 7 [file LSA-2021-01104_SdataF5.pdf]
